# Musculoskeletal Conditions and Secondary Cardiovascular Morbidity Increase Veterans’ Rehabilitation and Orthopaedic Service Utilization

**DOI:** 10.7759/cureus.27139

**Published:** 2022-07-22

**Authors:** Jessica C Rivera, Megan E Amuan, Mary Jo Pugh

**Affiliations:** 1 Orthopaedic Surgery, Louisiana State University Health Sciences Center, New Orleans, USA; 2 Surgery, Southeastern Louisiana Veterans Healthcare System, New Orleans, USA; 3 Statistics, VA Salt Lake City Veterans Health Care System, Salt Lake City, USA; 4 Medicine/Epidemiology, University of Utah School of Medicine, Salt Lake City, USA

**Keywords:** veterans health, service-connected disability, musculoskeletal disability, cardiac comorbidity, rehabilitation services

## Abstract

Background

Musculoskeletal conditions often affect patients’ mobility and ability to participate in health behaviors such as exercise, potentially affecting their systemic health. The purpose of this research is to determine how frequently cardiac-related comorbidities present in a veteran population with musculoskeletal service-connected disability and how this affects musculoskeletal health care utilization.

Methodology

A retrospective cohort of Iraq and Afghanistan Veterans who received a Veterans Affairs (VA) disability determination for service-connected musculoskeletal disability were categorized according to the diagnosis of cardiac comorbidity including diabetes mellitus, hyperlipidemia, hypertension, and obesity, and atherosclerosis disease documented by ICD-9 codes in the VA administrative data. Among veterans with musculoskeletal service-connected disability, logistic regression was modeled to determine if musculoskeletal clinic utilization was associated with also having a cardiac comorbid condition.

Results

Veterans with musculoskeletal disability had a comorbid cardiac disorder 43% of the time. Post-traumatic arthritis was the only musculoskeletal condition positively associated with comorbid cardiac conditions. Veterans with comorbid cardiac diagnoses had 26-37% higher odds of receiving care by physical and occupational therapy, physical medicine, and orthopaedic surgery clinics compared to veterans without comorbid cardiac disease.

Conclusions

Veterans in this cohort with musculoskeletal service-connected disability, plus cardiac conditions had greater clinic use for musculoskeletal and rehabilitation services compared to those without cardiac conditions. These results have implications for the rehabilitation and other health service needs of a new generation of veterans.

## Introduction

Musculoskeletal conditions are highly prevalent in military and veteran populations, associated with both combat deployment and standard military training [1,2]. Musculoskeletal conditions are the leading cause of medical separation from the military, extrapolating to high rates of musculoskeletal-related disability [3,4]. Musculoskeletal disorders such as arthritis and spine/back conditions are among the most common conditions treated by the Veterans Affairs (VA) health care system [5]. While musculoskeletal conditions alone result in a high burden of health care and disability, these conditions have also been shown to be associated with subsequent or increased severity of other comorbidities. This is specifically true of osteoarthritis and has an association with cardiovascular disease [6-8].

Because musculoskeletal conditions are common in military/veteran populations and musculoskeletal conditions have previously been associated with cardiovascular comorbid conditions, veterans with musculoskeletal-related disability may also have pertinent cardiovascular conditions that affect musculoskeletal care. Recovery from or the care of musculoskeletal conditions generally involves orthopaedic and rehabilitation services. Cardiovascular conditions which limit functional capacity may limit a patient’s ability to participate in these services such as physical therapy. Such patients may require a longer duration of therapy as they attempt to achieve the gains of that therapy. The purpose of this research is to determine how frequently cardiac-related comorbidities present in veteran populations with and without musculoskeletal service-connected disability. Secondly, we will compare musculoskeletal care services between veterans with musculoskeletal service-connected disability plus comorbid cardiovascular disease versus those with no comorbid cardiovascular conditions. We hypothesize that cardiovascular conditions are common in veterans with musculoskeletal service-connected disability and that veterans with musculoskeletal service-connected disability plus comorbid cardiovascular conditions will utilize more rehabilitation and musculoskeletal-related clinical services.

## Materials and methods

A retrospective cohort of veterans who received a disability determination for a service-connected disability by the Veterans Benefits Administration (VBA) before February 25, 2015, and received VA care in the fiscal year 2014 (October 1, 2013-September 30, 2014) and at least one other year between 2002-2013 was identified. This population included individuals deployed in support of the Operations Enduring Freedom or Operation Iraqi Freedom/Operation New Dawn as identified in the OEF/OIF/OND Roster from VA Environmental Epidemiology Service.

When a new veteran enters the VA, s/he is evaluated for medical conditions that are related to his or her military service that could result in prolonged disability. These conditions, or “service-connected disabilities,” are described using the Veterans Affairs Schedule for Rating Disabilities which essentially groups similar diagnosis codes into any number of corresponding “disability codes” [9]. These VA disability codes are included in each veteran’s data stored by the VA in the Veterans Services Network Corporate Master File (VETSNET). The disability codes abstracted from the VETSNET file were used to categorize veterans with and without musculoskeletal-related service-connected disability.

Comorbid conditions included hyperlipidemia, hypertension, atherosclerotic disease, congestive heart failure, and a history of myocardial infarction. These conditions were identified using algorithms validated in prior publications utilizing Medicare administrative data with International Classification of Diseases, 9th Revision (ICD-9) codes assigned at two outpatient clinical encounters at least seven days apart or one inpatient encounter [10-11]. We also identified healthcare utilization for outpatient rehabilitation (physical therapy, occupational therapy, prosthetics, physical medicine) using VA clinic codes for these clinics. Demographic characteristics included in this study were obtained from VA inpatient and outpatient data; missing data was supplemented using data from the OEF/OIF/OND Roster, which was derived from Department of Defense (DoD) demographic data. Age was analyzed as a continuous variable or ordinal variable with levels of 20-29 years, 30-39 years, 40-49 years, 50-59 years, and 60 or more years. The service component was classified as Active Duty, National Guard, or Reserves. Sex was defined as male or female. Race/ethnicity was defined as White, Black, Hispanic, and Other/unknown. Marital status was classified as married and not married.

First, the frequencies of cardiac-related conditions were compared between veterans with and without musculoskeletal service-connected disability, and differences were examined using a chi-squared statistic. Next, in veterans with one or more musculoskeletal service-connected disabilities, logistic regression was performed to determine if the presence of cardiac comorbidity was associated with a specific one or group of musculoskeletal service-connected disability or demographic. Finally, among veterans with musculoskeletal service-connected disability, logistic regression was performed to determine if musculoskeletal care utilization (physical therapy services, physical medicine, and rehabilitation services, orthopaedic surgery) was associated with the presence of cardiac comorbidity, a specific one or group of musculoskeletal service-connected disability, or demographic. Sequential models were compared with the addition of potentially confounding predictors and model fit was assessed using log-likelihood and Bayesian Information Criteria (BIC). Regression results are reported in odds ratios and 95% confidence intervals. All analyses were performed using Stata 14.0 (StataCorp, College Station, USA).

## Results

Veterans with and without musculoskeletal disability

383,757 veterans were identified for inclusion: 347,643 veterans with musculoskeletal service-connected disability and 36,114 with no musculoskeletal disability. Veterans with musculoskeletal disability had a comorbid cardiac disorder in 149,564 cases (43.0%). Veterans without musculoskeletal disability had a comorbid cardiac condition in 14,763cases (40.8%). Differences in frequency of individual comorbid conditions were not clinically significant between veterans with and without musculoskeletal service-connected disability. 

Musculoskeletal disabled veterans with and without cardiac conditions

The remaining analysis examined only veterans with musculoskeletal service-connected disability (n=347,643). Table 1 demonstrates the demographics of the veterans with musculoskeletal service-connected disability with and without a cardiac comorbid condition. The two groups were statistically different in each category though likely clinically meaningful difference show that veterans with cardiac conditions were older, less likely to have served on active duty status, less likely to be white, more likely to be obese, and more likely to be married.

**Table 1:** Demographics and column percentages of veterans with musculoskeletal service-connected disability with and without a cardiac-related comorbid condition (n = 347,643)

		Cardiac (n, %)	No Cardiac (n, %)	
Sex (male)		136,497 (91.3%)	166,913 (84.3%)	
Age (Mean ± SD)		42.8±9.9 years	34.5 ± 8.1 years	
Service Component				
	Active Duty	89,638 (59.9%)	150,436 (76.0%)	
	National Guard	38,128 (25.5%)	29,703 (15.0%)	
	Reserves	21,798 (14.6%)	17,941 (9.1%)	
Race/Ethnicity				
	White	93,229 (62.3%)	135,953 (68.6%)	
	Black	33,688 (22.5%)	33,722 (17.0%)	
	Hispanic	18,697 (12.5%)	23,243 (11.2%)	
	Other/Unknown	3,950 (2.6%)	5,161 (2.6%)	
Obese		54,504 (36.4%)	28,796 (14.5%)	
Married		91,688 (61.3%)	82,417 (41.6%)	

When testing for association between various service-connected disabilities and cardiac diagnoses, logistic regression indicates that arthritis is positively associated with cardiac disease in models accounting for only service-connected conditions. Degenerative arthritis service-connected disability results in 1.4 times greater odds for cardiac disease; and post-traumatic arthritis (PTA) 1.8 times greater odds (Models 1 and 2, p < 0.0001). When accounting for spine conditions, amputations, and any upper extremity or lower extremity service-connected disability, both specific arthritis conditions remain positively associated while spine conditions, back conditions, and lower extremity conditions resulted in statistically significant lower odds (p < 0.0001 for all). Only upper extremity conditions were not associated (Model 2, p = 0.125). The final model accounting for sex, age, and race/ethnicity is the best fitting model by log-likelihood and BIC. In this model, Age is the most strongly associated with an increased odds of cardiac diagnoses. In the controlled model, degenerative arthritis specifically is no longer positively associated but post-traumatic arthritis remains so. Amputation was not associated with cardiac diagnosis status (p = 0.264) and compared to White subjects, subjects with Other/unknown race/ethnicity were not associated with cardiac disease status (p = 0.392). The remaining associations were all statistically significant (p < 0.0001). Table 2 demonstrates the full regression results.

**Table 2:** Logistic regression testing for associations between cardiac disease status and service-connected disability (SCD)/demographics (n = 347,643) PTA: post-traumatic arthritis; BIC: Bayesian Information Criteria

	Model 1		Model 2		Model 3	
	OR	95% CI	OR	95% CI	OR	95%CI
Osteoarthritis SCD	1.49	1.44-1.53	1.46	1.42-1.50	0.88	0.85-0.91
PTA SCD	1.89	1.84-1.95	1.84	1.79-1.89	1.08	1.05-1.12
Spine/Back SCD			0.98	0.97-0.99	0.93	0.91-0.95
Amputation SCD			0.91	0.83-0.99	1.18	1.07-1.30
UpperEx SCD			1.03	1.01-1.04	0.94	0.93-0.96
LowerEx SCD			0.82	0.79-0.82	0.88	0.86-0.89
Sex (ref: Male)					0.44	0.43-0.45
Age (ref: 20-29years)						
30-39 years					2.40	2.34-2.46
40-49 years					6.42	6.25-6.59
50-59 years					12.23	11.84-12.63
60 or more years					23.18	21.74-24.72
Race (ref: White)						
Black					1.24	1.22-1.27
Hispanic					1.11	1.08-1.14
Other/Unknown					0.97	0.93-1.02
Obese					3.20	3.14-3.26
Married					1.13	1.11-1.15
Log likelihood	-236,159		-235,763		-195,332	
BIC	472,357		471,617		390,919	

The proportion of veterans seen in physical and occupational therapy, physical medicine, and orthopaedic surgery clinics was consistent over a span of six years despite fluctuations in the number of veterans receiving care in any given year. Additionally, veterans with comorbid cardiac were consistently more likely to be seen in these clinics compared to veterans without comorbid cardiac disease. Physical and occupational therapy was utilized during at least one year of care by 41.8% of veterans with no cardiac conditions and 52.2% of veterans with cardiac conditions. Physical Medicine and Rehabilitation professionals saw 16.1% of veterans without cardiac conditions and 20.6% of veterans with. Orthopaedic surgeons saw 38.1% of veterans without cardiac conditions and 47.0% of veterans with cardiac comorbidity. All clinics combined, 60.6% of veterans without cardiac conditions utilized one or more musculoskeletal clinics between 2010 and 2015 while 70.3% of veterans with comorbid cardiac conditions sought care in that timeframe from musculoskeletal clinics.

Cardiac comorbidity was positively associated with musculoskeletal clinic use, resulting in 22-37% greater odds per clinic. Veterans with PTA utilized clinics more than veterans with degenerative osteoarthritis; however, both arthritic groups were more likely to see an orthopaedic surgeon than a rehabilitation professional. Veterans with amputations had the greatest odds relative to other variables of musculoskeletal clinic use. Older age tended to result in greater odds of orthopaedic clinic use; however, despite the increasing frequency of comorbid cardiac conditions as veterans become older, greater rehabilitation service utilization was not associated with increasing age. Table 3 demonstrates regression results testing for association between conditions and demographics and the use of musculoskeletal-related clinics. 

**Table 3:** Logistic regression results in odds ratios and 95% confidence intervals for service-connected disability (SCD) and demographics and associations with musculoskeletal clinics in year 2014 (n = 347, 643) PTA: post-traumatic arthritis

	Physical/Occupational Therapy	Physical Medicine	Orthopaedic Surgery	All MSK Clinics
Cardiac Comorbidity	1.37 [1.35-1.39]	1.23 [1.20-1.26]	1.22 [1.20-1.24]	1.37 [1.34-1.39]
Osteoarthritis SCD	1.02 [0.99-1.05]	1.01 [0.97-1.05]	1.27 [1.24-1.31]	1.15 [1.11-1.18]
PTA SCD	1.16 [1.13-1.20]	1.15 [1.11-1.19]	1.42 [1.38-1.46]	1.30 [1.26-1.34]
Spine/Back SCD	1.48 [1.46-1.50]	1.60 [1.57-1.63]	0.93 [0.92-0.95]	1.31 [1.29-1.33]
Amputation SCD	2.85 [2.61-3.11]	3.29 [3.01-3.59]	1.86 [1.71-2.02]	3.88 [3.44-4.37]
UpperEx SCD	1.14 [1.13-1.16]	1.13 [1.10-1.15]	1.24 [1.22-1.26]	1.19 [1.17-1.21]
LowerEx SCD	1.04 [1.02-1.05]	0.98 [0.97-1.00]	1.37 [1.35-1.39]	1.10 [1.08-1.13]
Sex (ref: Male)	1.17 [1.15-1.20]	1.07 [1.04-1.10]	1.06 [1.04-1.08]	1.10 [1.08-1.13]
Age (ref: 20-29)				
30-39 years	1.09 [1.07-1.11]	1.11 [1.08-1.13]	1.15 [1.13-1.17]	1.11 [1.09-1.14]
40-49 years	0.99 [0.97-1.01]	0.97 [0.94-1.00]	1.23 [1.20-1.26]	1.04 [1.01-1.06]
50-59 years	0.97 [0.94-1.00]	0.93 [0.90-0.97]	1.34 [1.30-1.38]	1.00 [0.98-1.04]
60 or more years	0.85 [0.81-0.90]	0.89 [0.84-0.95]	1.32 [1.26-1.39]	0.90 [0.85-0.94]
Race (ref: White)				
Black	1.21 [1.19-1.23]	1.17 [1.14-1.20]	1.18 [1.16-1.21]	1.21 [1.19-1.24]
Hispanic	1.16 [1.13-1.18]	1.94 [1.89-1.99]	1.21 [1.18-1.23]	1.30 [1.27-1.33]
Other/Unknown	0.79 [0.75-0.82]	0.83 [0.78-0.88]	0.90 [0.86-0.94]	0.82 [0.79-0.86]
Obese	1.43 [1.41-1.46]	1.39 [1.35-1.41]	1.32 [1.30-1.34]	1.46 [1.43-1.48]
Married	0.95 [0.94-0.97]	0.95 [0.93-0.97]	0.96 [0.95-0.98]	0.95 [0.93-0.97]

## Discussion

Veterans with and without musculoskeletal service-connected had non-clinically significant differences in cardiac conditions. Veterans with musculoskeletal service-connected disability and cardiac conditions were older, less likely to have served on active duty status, more likely to be black, more likely to be obese, and married compared to veterans with no cardiac conditions. Age was the most highly associated variable with cardiac conditions. Cardiac comorbidity in veterans with musculoskeletal service connection was positively associated with musculoskeletal clinic use.

Veterans in this cohort with a musculoskeletal service connection did not have a clinically meaningful, albeit statistically different, frequency of cardiac diagnoses compared to veterans without a musculoskeletal service connection. Several reports in the literature suggest the opposite, specifically for degenerative and rheumatoid arthritis. A recent meta-analysis suggests that the link between arthritis and cardiovascular disease is substantial [6]. Other reports have found an association between disease severity for each condition: arthritic comorbid to cardiovascular disease resulted in more severe vascular pathology and high mortality [8,12]. These studies were performed with older subjects which may explain part of the difference between the current literature and the findings herein. In fact, regression modeling indicates that age is the most prominent variable associated with comorbid cardiac conditions in musculoskeletal service-connected veterans; however, the mean age of even our musculoskeletal plus cardiac condition veterans was only 42.8 years of age. Because veterans do have a higher prevalence of osteoarthritis compared to civilian counterparts, the impact of comorbid cardiovascular disease and arthritis will likely become more evident and more prominent as these veterans age [13-15]. 

As opposed to degenerative arthritis, post-traumatic arthritis remained positively associated with cardiac diagnoses after accounting for demographics in the regression modeling. This is an important distinction between a service connection for degenerative arthritis because post-traumatic arthritis would presumably be directly attributable to an injury event and typically occurs in patients much younger than does degenerative arthritis [16,17]. The ensuing pathophysiology is thought to be more inflammatory in post-traumatic cases just as cardiovascular disease is increasingly understood to be associated with inflammation. Although much of the literature on arthritis fails to distinguish between degenerative causes and post-traumatic causes, this is the first report to suggest an association between post-traumatic arthritis and cardiac diagnoses. For veterans and military personnel at high risk for joint injury and relatively greater risk for post-traumatic causes of arthritis, this could have an impact on the cardiovascular health of specific generations of combat veterans [18-21].

Veterans in this cohort with musculoskeletal service-connected disability plus cardiac conditions did have greater clinic use for musculoskeletal services compared to those without cardiac conditions. This is consistent with prior literature that the functional impairment due to musculoskeletal conditions, specifically arthritis, is impacted by comorbid cardiovascular disease [7,22]. This may be because comorbid conditions that contribute to cardiac dysfunction can interfere with a patient’s ability to participate in rehabilitation services for the duration and intensity prescribed to treat the musculoskeletal condition. Also, such conditions themselves cause functional impairment; and the association to these clinic uses may be more of a reflection of the overlap between practice patterns for prescribing therapy and rehabilitation services. However, the association extends also to orthopaedic surgery clinic use which may reflect the increase in musculoskeletal conditions that come with age. As such the test for association between clinic use and age, arthritis specifically, and cardiac conditions are not entirely independent. However, this does not lessen the impact on health care expenditures from cardiac conditions in musculoskeletal patients. Cardiac comorbidity resulted in the highest odds of all musculoskeletal clinic use second only to veterans with amputations, but cardiac conditions in veterans are far more prevalent than amputations.

One challenging finding of these results is that clinic utilization for both degenerative and post-traumatic arthritis is predominantly of orthopaedic surgery services as opposed to rehabilitation services. This is in direct contradiction to how arthritis, specifically of the knee, should be treated. The American Academy of Orthopaedic Surgeons clinical practice guideline for treating knee arthritis strongly supports strengthening, low-impact aerobic exercise, and neuromuscular education in lieu of invasive procedures such as injection therapy or non-arthroplasty surgical treatments [23]. While these data are not able to indicate if orthopaedic surgery clinic utilization leads to procedures, they do indicate that the strongest recommendation for rehabilitation techniques is not evident in this veteran population. This finding is particularly evident in older veterans who could theoretically benefit from rehabilitation services for their increasing frequency of arthritis as well as increased frequency of cardiovascular disorders compared to younger veterans.

This research has a number of limitations. First, these comparison groups are delineated due to service-connected disability rather than diagnosis codes. As such, individuals who receive care for musculoskeletal disorders or for cardiac disorders but were not designated as having a service-connected disability for musculoskeletal conditions may be misclassified. This could cause over or underestimation of the tested associations and clinic utilization. Secondly, this analysis does not represent changes in utilization with over time (e.g., more veterans entering the VA system) or cumulatively over the entire span of the VETSNET data. We have, however, chosen the latest time point for which the VETSNET data is complete and required subjects to have care within the VA during at least one preceding year in order to maximize the number of subjects available for analysis while minimizing the number of individuals who may be evaluated once by the VA for service connection purposes but not receive any additional care. As such, we hope to provide the most recent estimates which are most accurately represented for care utilization in the VA today. Third, the comparison groups are categorized based on presence or absence of service-connected disability for musculoskeletal or diagnoses of at least one cardiac-related condition and we did not attempt to account for specific overlapping conditions of multiple cardiac conditions’ cumulative effect on health care.

## Conclusions

In summary, a strong difference between veterans with and without musculoskeletal service-connected disability and cardiac comorbidities was not identified in this cohort. However, the impact of musculoskeletal and cardiac conditions together on the veterans’ population as a whole is tremendous. This is because of the high frequency of musculoskeletal conditions in veterans, an association between post-traumatic arthritic and cardiac conditions, and an association between comorbid cardiac conditions and increased musculoskeletal clinic use. As this generation of combat veterans ages, one can expect this impact to be increasingly pronounced.
